# Radiotherapy Combined with Immune Checkpoint Inhibitor on Murine Fibrosarcoma and a Narrative Review of Clinical Studies

**DOI:** 10.3390/cimb48010020

**Published:** 2025-12-24

**Authors:** Wonwoo Kim, Hyunkyung Kim, Won Il Jang, Mi Sook Kim, Sun Hyun Bae

**Affiliations:** 1 AI Institute for Drug Discovery, Korea Pharmaceutical and Bio-Pharma Manufacturers Association, Seoul 06666, Republic of Korea; wonwookim@gmail.com; 2 Department of Radiation Oncology, Korea Institute of Radiological & Medical Sciences, Seoul 01812, Republic of Korea; hk0811@kirams.re.kr (H.K.); zzang11@kirams.re.kr (W.I.J.); 3 Department of Radiation Oncology, Soonchunhyang University College of Medicine, Bucheon 14584, Republic of Korea

**Keywords:** radiotherapy, immunotherapy, fibrosarcoma, mouse model, soft tissue sarcoma

## Abstract

**Purpose:** The objective of this study was to evaluate the synergistic effect of combining an immune checkpoint inhibitor (ICI) with radiotherapy (RT) on murine fibrosarcoma and to conduct a narrative review of clinical studies on soft tissue sarcoma (STS). **Materials and Methods:** Forty male C3H mice (aged 5 weeks) were injected intramuscularly with 2 × 10^5^ FSaII cells into the right thigh and randomly assigned to four groups: (1) control; (2) RT; (3) InVivoMab™ (CD279) (mouse anti-PD-1 antibody) (ICI group); and (4) combined treatment with InVivoMab™ (CD279) and RT (combination group). On day −1, ICI was administered intraperitoneally. On day 0, RT (10 Gy, single fraction) was delivered locally to the tumors in the right hind limb. Subsequently, ICI was injected twice weekly (a total of 8 times). On day 26, all mice were euthanized, and the results were analyzed. In addition, a narrative review was conducted to identify clinical evidence. **Results:** On day 26, mean gross tumor volumes were 3578.13 ± 407.32 mm^3^ in the control group, 1995.72 ± 970.46 mm^3^ in the RT group, 2729.96 ± 286.47 mm^3^ in the ICI group, and 1007.92 ± 197.36 mm^3^ in the combination group. Gross tumor growth delay was most pronounced in the combination group. Moreover, the TUNEL assay demonstrated a significant increase in apoptosis in the combination group. Analysis of the underlying immune system revealed significantly higher expression of CD4+, CD8+, and IFN-γ in the combination group. The literature search identified only 12 case reports and 3 prospective studies involving patients with STS treated with the combined treatment of ICI and RT, suggesting potential synergism with acceptable toxicity. **Conclusions:** The current study demonstrated a synergistic effect of combining an ICI with RT in murine fibrosarcoma. There was limited data in the clinical setting. Further investigations are warranted to determine the optimal combination strategy of ICI and RT for STS.

## 1. Introduction

Soft tissue sarcoma (STS) is a rare malignancy originating from mesenchymal tissue and accounts for approximately 1% of adult cancers [1]. It is further subclassified into nearly 70 subtypes, each characterized by distinct morphologic features [2]. Although the primary treatment for STS is surgical excision with adequate margins, the rarity of the disease and its variable biological and clinical behavior necessitate a highly coordinated multidisciplinary approach [3]. External beam radiotherapy (EBRT) is considered a standard treatment for localized STS and can be administered either preoperatively or postoperatively [4,5,6]. Furthermore, with advancements in RT technology, the role of EBRT has expanded. Stereotactic body radiotherapy (SBRT), which delivers a high-dose RT to the tumor in a single or few fractions, has shown promising results with high local control rates for both primary and metastatic STS lesions [7,8,9]. Conventional chemotherapy, including anthracycline-based regimens, is recommended for advanced and metastatic STS [10,11]. However, even with combined treatment strategies, approximately 50% of patients with STS experience disease recurrence and metastases [12].

Recently, the integration of immunotherapy into traditional treatment regimens has brought a paradigm shift in oncology. Among immunotherapies, immune checkpoint inhibitors (ICIs)—which block molecules such as programmed cell death protein 1 (PD-1) or programmed cell death protein ligand 1 (PD-L1)—have become a standard treatment for various malignancies, significantly improving patient survival. PD-1 was first identified in 1992 in the 2B4-11 (murine T-cell hybridoma) and the interleukin-3-deprived LyD9 (murine hematopoietic progenitor cell line) [13]. PD-1 is an inhibitor of both adaptive and innate immune responses, and its expression is induced in activated T cells, natural killer cells, B lymphocytes, macrophages, dendritic cells (DCs), and monocytes [14]. PD-1 is also upregulated in tumor-specific T cells. It plays a key role in reducing the regulation of ineffective or harmful immune responses and maintaining immune tolerance through suppressing T-cell activity and promoting the differentiation of regulatory T cells [15]. However, this inhibitory mechanism can cause the dilation of tumor cells by disrupting protective immune responses [16]. Upon binding to its ligands PD-L1 or PD-L2, both members of the B7 family, signaling inhibits the active immune cells [17]. PD-L1 is constitutively expressed by macrophages, some activated T and B cells, DCs and certain epithelial cells, particularly under inflammatory conditions [18]. Tumor cells can also express PD-L1 as an adaptive immune mechanism to evade antitumor immunity [19]. The PD-1/PD-L1 pathway plays a crucial role in regulating T-cell activation, proliferation, and cytotoxic secretion in cancer, and PD-1/PD-L1 inhibitors demonstrated significant therapeutic efficacy across many cancers [20].

However, ICIs have shown limited efficacy in STS [11,21]. One potential strategy to enhance the therapeutic efficacy of ICIs in STS is their combination with RT. Although the primary mechanism of RT in cancer treatment is to induce DNA damage and thereby kill tumor cells, it is also known that RT modulates the immune system through multiple mechanisms that influence therapeutic outcomes [22]. RT can stimulate anti-tumor immune responses in several ways: (1) by enhancing major histocompatibility complex (MHC) class I surface expression, activating DCs, and promoting cross-presentation of tumor antigens that activate CD8+ T cells; (2) by increasing the release of chemokines and promoting margination and extravasation, which elevate the density of tumor-infiltrating lymphocytes; and (3) by upregulating FAS surface expression, leading to programmed cell death [23]. Based on these effects, RT has long been proposed to act synergistically with immunotherapy, enhancing local antitumor response in primary tumors and inducing systemic immune activation against distant tumors [24]. However, RT simultaneously trigger compensatory mechanisms that contribute to immunosuppression: (1) increasing PD-L1 expression on tumor cells and regulating the expression of multiple IC molecules on immune cells, thereby promoting tumor immune evasion and T-cell exhaustion; (2) enhancing regulatory T cells (Tregs), which suppress both adaptive and induced immune responses; and (3) inducing lymphopenia and immune cell depletion [25,26]. These counteracting effects of RT may represent a major obstacle to realizing the full synergistic potential of RT and ICIs for cancer therapy in the real world [27].

Historically, larger RT fields using two-dimensional or three-dimensional conformal RT techniques encompassed the substantial volumes of bone marrow, resulting in marked lymphocyte depletion and reinforcing the notion that RT is generally immunosuppressive [28]. However, modern RT techniques, including SBRT, have markedly reduced treatment volumes, enabling the delivery of higher tumoricidal doses while minimizing radiation exposure to surrounding normal tissues [29,30]. Moreover, accumulating preclinical and clinical evidence indicates that RT can activate the immune system and induce immunogenic cell death [31]. These findings support the potential of the synergistic interaction between ICI and RT.

Therefore, the current study evaluated the synergistic effect of combining an ICI with RT in a murine fibrosarcoma model and conducted a narrative review to collect the available clinical evidence in humans.

## 2. Materials and Methods

### 2.1. Tumors and Mice

All experiments were conducted in accordance with the animal experimental protocol (KIRAMS 20170078) approved by the Korea Institute of Radiological and Medical Sciences.

The FSaII fibrosarcoma cell line of C3H mice, derived from a soft tissue sarcoma, was provided by Dr. Chang W. Song at the University of Minnesota. Experimental procedures were established in our laboratory as previously described [32]. Stock tumor cells were thawed and cultured in RPMI-1640 media (Cat. LM011-04, Welgene, Republic of Korea) supplemented with 10% fetal bovine serum (Cat. S001-07, Welgene, Republic of Korea) and antibiotics (Cat. LS202-02, Welgene, Republic of Korea) under humidified conditions at 37 °C in a 5% CO_2_ atmosphere. Tumor cells in the exponential growth phase were harvested by trypsinization, and approximately 2 × 10^5^ viable tumor cells were injected subcutaneously into the right hind limbs of 5- to 6-week-old male C3H mice. Tumor volume (V, mm^3^) was calculated using the Vernier caliper formula, V = 0.523 × (shortest diameter)^2^ × (longest diameter). Tumors were utilized for experiments when their diameters reached 7–8 mm.

Five-week-old C3H mice were purchased from Orient Bio Co. (Seongnam, Republic of Korea) and allowed to acclimate to the new environment for one week before the experiments. The animal room was maintained at 22 ± 3 °C and 50 ± 20% relative humidity. Water and food (Purina, St. Louis, MO, USA) were provided ad libitum.

### 2.2. Treatment

For the tumor growth delay study, tumor-bearing mice were randomly divided into four groups (10 mice per group) as follows: (a) no treatment (control group); (b) irradiation (RT group); (c) anti-PD-1 antibody (InVivoMab group); and (d) combined treatment with anti-PD-1 antibody and irradiation (InVivoMab+RT group).

**Figure 1:**
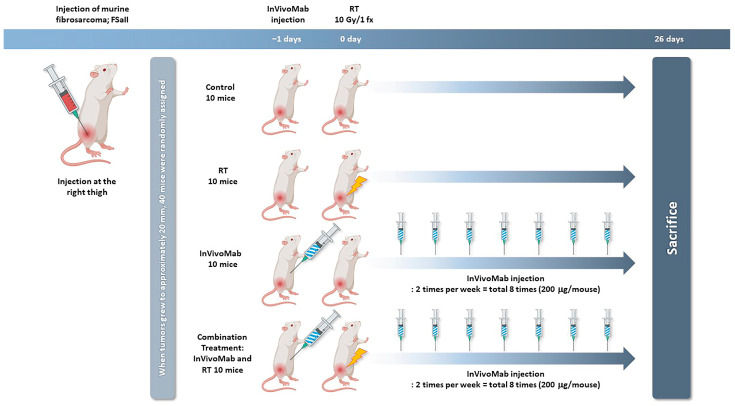
Experimental design.

Mice were lightly anesthetized by intramuscular injection of tiletamine/zolazepam (Virbac Zoletil™ 50, Virbac Laboratories, Carros, France), and the tumor-bearing legs were locally irradiated with a single dose of 10 Gy using a ^60^Co unit (Thermatron 780, Atomic Energy of Canada Limited, Chalk River, ON, Canada) at a dose rate of 1.3 Gy/min. InVivoMab anti-mouse PD-1 (CD279, Cat. #BE0146, BioXcell, Lebanon, NH, USA) was purchased from BioXcell (USA). InVivoMab anti-PD-1 antibody was intraperitoneally injected at a dose of 10 mg/kg one day before irradiation and then twice weekly for three weeks after RT, for a total of eight injections. Tumor diameters were measured with calipers every other day until day 26 after the start of treatment. On day 26, mice were euthanized by carbon dioxide (CO_2_) inhalation. Figure 1 illustrates the experimental design for all treatment groups.

### 2.3. Apoptosis Assay

On day 26 after treatment, tumors were excised and fixed in neutral-buffered formalin. After paraffin embedding, tumors were sectioned at a thickness of 4 µm, mounted on silane-coated slides, deparaffinized, and stained using the ApopTag^®^ Peroxidase In Situ Apoptosis Detection Kit (Cat. S7100, Merck Millipore, Billerica, MA, USA). Apoptotic cells positive for terminal deoxynucleotidyl transferase-mediated dUTP-nick end labeling (TUNEL) staining were counted at 400× magnification according to the manufacturer’s instructions. TUNEL-positive cells were defined as apoptotic only when they displayed typical apoptotic morphology.

### 2.4. Immunohistochemical Staining

Tumor tissue sections were mounted on silane-coated slides, deparaffinized, and boiled for 10 min in 0.01 M citrate buffer (pH 6.0) for antigen retrieval. The sections were then incubated overnight at 4 °C with solutions containing appropriate concentrations of antibodies against CD4 (Cat.NBP1-19371, Novus Biologicals, Centennial, CO, USA), CD8 (Cat.ab4055, Abcam, Waltham, MA, USA), and interferon gamma (IFN-γ, Cat. 15365-1-AP, Proteintech, Rosemont, IL, USA). The following day, the sections were washed three times with phosphate-buffered saline (PBS) and incubated with peroxidase reagent and anti-mouse IgG (Cat. MP-7402, ImmPRESS™, Vector, Laboratories, Burlingame, CA, USA) for 20 min. After three additional washes with PBS, peroxidase-binding sites were stained using diaminobenzidine (Cat. SK-4100, DAB, Vector, Laboratories, Burlingame, CA, USA), counterstained with Mayer’s hematoxylin (Cat. ab220365, Abcam, Waltham, MA, USA) and examined under a light microscope.

### 2.5. Study Search

A literature search was conducted using the PubMed database on 11 May 2025. The search strategy was as follows: (“sarcoma” OR “soft tissue sarcoma” OR “fibrosarcoma”) AND (“immunotherapy” OR “immune checkpoint inhibitor”) AND (“radiotherapy” or “radiation therapy”). We included clinical studies involving human subjects with STS treated with RT and ICI, published in English with no time restrictions. Studies were excluded if combined treatment was applied to (1) pediatric patients; (2) patients with special histologic subtypes (Ewing sarcoma, rhabdomyosarcoma, or Kaposi sarcoma); (3) patients with secondary STS following RT or allogeneic stem cell transplantation; and (4) patients with primary brain STS. In addition, reference lists of included articles were reviewed to identify other eligible studies. Two independent reviewers identified eligible studies and extracted data from the selected publications.

### 2.6. Statistical Analysis

All results are expressed as means ± standard error. Statistical analyses were performed using one-way analysis of variance (one-way ANOVA, SPSS software [version 19.0; IBM Corp., Armonk, NY, USA]), followed by appropriate post hoc tests to assess differences between groups. A *p*-value less than 0.05 was considered statistically significant.

## 3. Results

### 3.1. Tumor Growth

Treatment was initiated when FSaII tumor volumes in C3H mice reached 111–141 mm^3^. Tumor growth was monitored across the four groups over a 26-day observation period. Final mean tumor volumes were 3578.13 ± 407.32 mm^3^ in the control group, 1995.72 ± 970.46 mm^3^ in the RT group, 2729.96 ± 286.47 mm^3^ in the InVivoMab group, and 1007.92 ± 197.36 mm^3^ in the InVivoMab+RT group. Compared with the control, tumor volumes were reduced by approximately 44% with RT, 24% with InVivoMab, and 72% with the combination treatment. These findings indicate that, while each monotherapy had limited efficacy in this aggressive tumor model, the combination therapy achieved the most pronounced tumor suppression.

**Figure 2:**
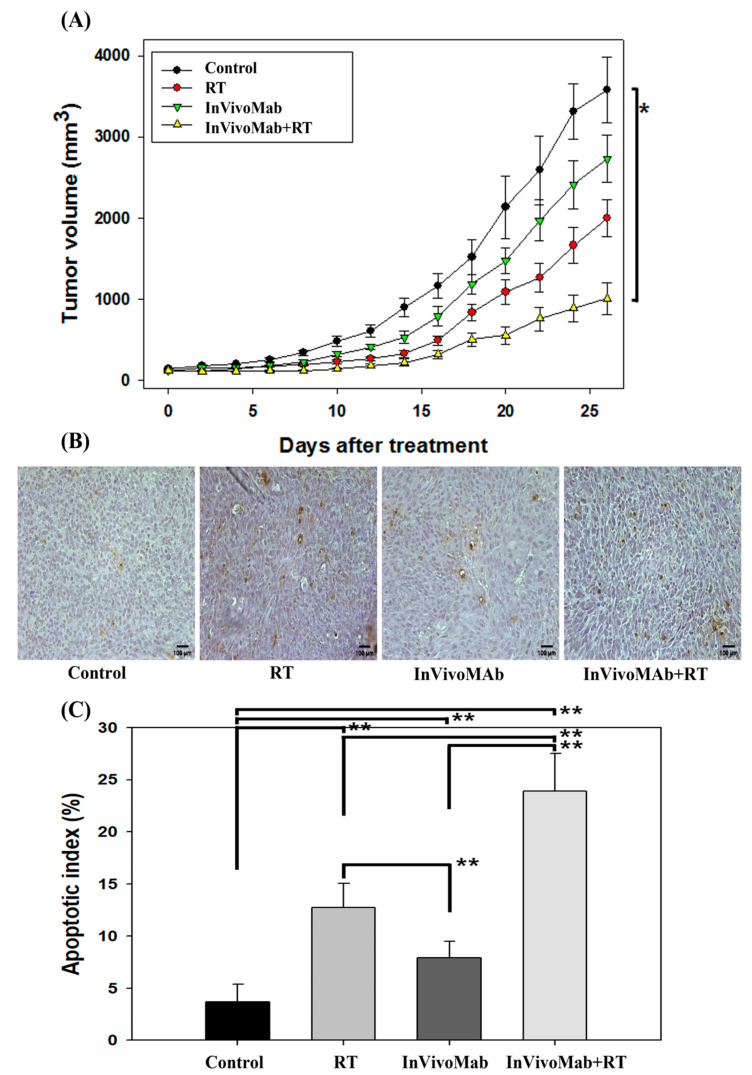
(**A**) FSaII tumor growth delay according to time after treatment in the four experimental groups. Mean volume of 10 tumors ± standard error is shown. (**B**) TUNEL assay for excised FSaII tumor tissues after the four experimental treatments. (**C**) Apoptotic index (%) after treatments. The number of apoptotic cells (total of 1000 nuclei) in 10 fields randomly selected in each of three sections per tumor was obtained. Mean of 10 tumors ± standard error is shown. Asterisk indicates significant difference between groups: * means *p* < 0.05; ** means *p* < 0.01.

Pairwise comparisons revealed statistically significant differences between the combination group and both monotherapy groups (*p* < 0.05). The InVivoMab+RT group consistently maintained lower tumor volumes throughout the study, as illustrated in Figure 2A. These results suggest a synergistic interaction between RT and ICI, leading to enhanced and sustained tumor control.

### 3.2. Apoptosis

Apoptosis induction in FSaII tumors following various treatments is shown in Figure 2. Representative TUNEL-stained sections for each treatment group are presented in Figure 2B and the corresponding percentage of apoptotic cells (apoptotic index) is presented in Figure 2C. In control tumors, 4 ± 1.70% of cells were apoptotic, whereas RT with 10 Gy increased the apoptotic index to 13 ± 2.36% (*p* < 0.01). Tumors treated with InVivoMab and InVivoMab+RT exhibited apoptotic indices of 8 ± 1.60% and 24 ± 3.63%, respectively (*p* < 0.01). The combination of InVivoMab and RT demonstrated the greatest induction of apoptosis among all groups.

### 3.3. Immunohistochemical Assay

**Figure 3:**
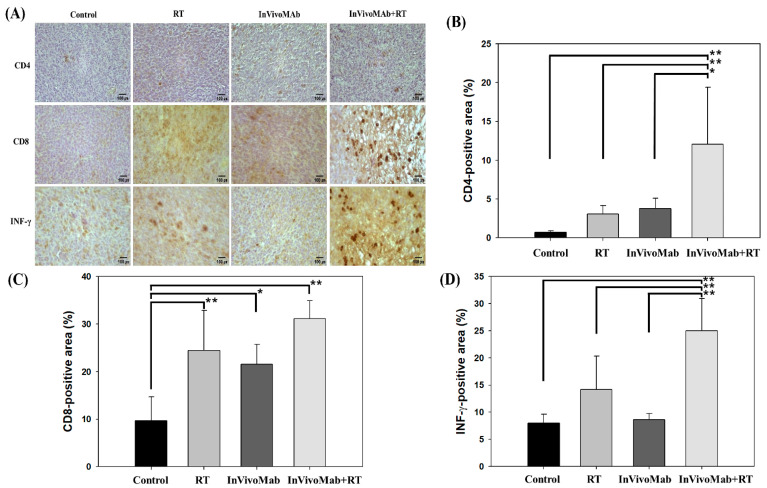
(**A**) Immunohistochemistry staining for excised FSaII tumor tissues after the four experimental treatments; (**B**) CD4, (**C**) CD8, and (**D**) IFN-γ. Asterisk indicates significant difference between groups: * means *p* < 0.05; ** means *p* < 0.01.

To characterize the immune response, the proportions of CD4+, CD8+ and IFN-γ–positive areas within the tumors were analyzed by immunohistochemistry. CD4+ and CD8+ staining were performed to evaluate the degree of T-cell infiltration and cytotoxic activity within the tumor microenvironment, whereas IFN-γ staining was conducted to assess the functional activation of tumor-infiltrating lymphocytes and the induction of a Th1-type immune response. The combination of InVivoMab and RT significantly increased tumor infiltration of CD4+ (12.05 ± 7.35%) and CD8+ (31.09 ± 3.82%) cells compared with the control groups (0.72 ± 0.21%, 9.68 ± 5.04%, respectively), indicating enhanced recruitment and activation of effector T cells. In parallel, the proportion of IFN-γ–positive areas increased markedly from 8 ± 1.64% in the control group to 25 ± 5.90% in the combination group, suggesting that combination therapy activates the inflammatory pathway and promotes the establishment of an antitumor immune microenvironment. Collectively, these findings demonstrate that PD-1 blockade with InVivoMab, particularly in combination with RT, promotes robust T-cell infiltration and functional activation within the tumor microenvironment, thereby enhancing antitumor immunity (Figure 3).

### 3.4. Narrative Review

A total of 379 studies were initially identified through PubMed database search. After screening, 13 studies were selected, and two additional studies were identified through cross-referencing. Thus, a total of 15 studies were included in this narrative review: 12 case reports (Table 1) and three clinical studies (Table 2) [33,34,35,36,37,38,39,40,41,42,43,44,45,46,47]. Various fractionation schemes were used among the case reports, with SBRT applied in six case reports. RT was administered with neoadjuvant, adjuvant, salvage, or palliative intent. Eleven case reports demonstrated promising outcomes with combined RT and ICI therapy without severe complications. Notably, three case reports involving undifferentiated pleomorphic sarcoma (UPS) treated with ICIs and SBRT reported long-term disease-free survival (DFS) exceeding two years [34,36,43]. Conversely, Chan et al. [40] reported rapidly progressive liver metastases after neoadjuvant ICI and RT followed by surgery for liposarcoma (LPS), raising the possibility of hyperprogression. All three clinical studies were prospectively conducted in the neoadjuvant setting. One was a randomized trial comparing RT and surgery versus RT, pembrolizumab, and surgery, while the remaining two were single-arm trials. UPS was the most common histologic subtype and conventional fractionation (50 Gy in 25 fractions) was applied. Local recurrence rates ranged from 0% to 11%, respectively. Two-year DFS and overall survival (OS) ranged 60–100% and 83–100%, respectively. Severe complications above grade 3 occurred in 17–56% of patients, respectively.

## 4. Discussion

The current study demonstrated that combined treatment with an ICI and RT produced the greatest inhibition of tumor growth and exhibited a synergistic effect in murine fibrosarcoma.

Few preclinical studies have focused on its immunomodulatory effects and the syner-gistic potential of combining RT with ICI in STS. Stone et al. [48] demonstrated that immunocompromised hosts required a much higher RT dose to achieve tumor control than immunocompetent hosts in a murine fibrosarcoma model, and that activation of the immune system through bacterial infection could enhance tumor control when administered with RT. Researchers at Duke University reported that neoadjuvant anti-PD-1 antibody therapy combined with RT (20 Gy in a single fraction) followed by surgery significantly improved local recurrence-free survival (LRFS) (*p* = 0.04) and DFS (*p* = 0.01), compared with surgery plus isotype control antibody in the p53/MCA sarcoma model [49]. The same group further observed that transplanted p53/MCA sarcomas in mice were cured by the synergistic effects of RT and anti-PD-1 antibody, whereas identical treatment failed in autochthonous sarcomas, which exhibited immunoediting, reduced neoantigen expression, and tumor-specific immune tolerance [50]. The authors suggested that the immune microenvironment of primary murine sarcomas resembles that of most human sarcomas, whereas transplanted sarcomas resemble highly inflamed human sarcomas, indicating that patients with sarcomas exhibiting an immune phenotype similar to transplanted tumors may derive the greatest benefit from ICI–RT combination therapy. The present study demonstrated that combined treatment with anti-PD-1 antibody and RT (10 Gy in a single fraction) elicited the most significant inhibition of tumor growth in FSaII fibrosarcoma. This effect was associated with a marked increase in CD4+ and CD8+ cell infiltration and elevated intratumoral IFN-γ expression in the combination group. IFN-γ is known to upregulate the expression of MHC molecules and PD-L1, thereby enhancing responsiveness to anti-PD-1 antibody therapy [51]. Collectively, these findings support that the combination of ICI and RT exerts a synergistic antitumor effect in murine fibrosarcoma.

ICIs are designed to block crucial regulatory pathways that suppress immune response, thereby enabling tumor-reactive T cells to mount an effective antitumor response [52]. Monoclonal antibodies targeting PD-1 (e.g., nivolumab, pembrolizumab, and camrelizumab), PD-L1 (e.g., atezolizumab and durvalumab), and cytotoxic T-lymphocyte antigen-4 (ipilimumab and tremelimumab) have been approved by the U.S. Food and Drug Administration (FDA) for the treatment of various cancers [53]. ICIs have been shown to prolong survival in patients with lung cancer, head and neck cancer, renal cell cancer, urothelial cancer, and malignant melanoma [54]. On the other hand, the efficacy of ICIs in STS appears limited. Most clinical trials have been small and heterogeneous, often conducted as basket trials including diverse sarcoma subtypes despite their distinct biological behaviors, thereby making it difficult to draw firm conclusions regarding their effectiveness in rare histologic subtypes [11]. Two systematic reviews and meta-analyses were subsequently performed to overcome the limitations of individual clinical studies. The first meta-analysis, published in 2021, reported a pooled objective response rate (ORR) of 14% (95% confidence interval [CI], 0.09–0.18) and a pooled disease control rate (DCR) of 55% (95% CI, 0.43–0.66) across 27 studies evaluating ICIs in STS [55]. Higher response rates were observed in alveolar soft part sarcoma (ASPS) and UPS, with ORRs of 35% and 20%, respectively, whereas limited activity was noted in leiomyosarcoma and LPS, with ORRs of 10% and 11%, respectively. An updated meta-analysis in 2025 yielded similar findings, showing a pooled ORR of 16% (95% CI, 0.12–0.21) and a pooled DCR of 64% (95% CI, 0.57–0.70) across 38 clinical studies [56]. Subtypes such as ASPS, angiosarcoma, and epithelioid sarcoma exhibited favorable ORRs exceeding 30%. Currently, only atezolizumab for ASPS has received FDA approval.

Combined treatment strategies may enhance the efficacy of ICIs in STS. Saerens et al. [55] reported that the combination of anti-PD-1 antibody and tyrosine kinase inhibitor (TKI) achieved the highest ORR of 24% (95% CI, 0.10–0.41) followed by anti-PD-1 antibody combined with chemotherapy (ORR = 20%; 95% CI, 0.06–0.38). Grade 3–5 complications occurred most frequently with anti-PD-1 antibody plus chemotherapy (74%; 95% CI, 0.06–0.85), and were less common in anti-PD-1 antibody plus TKI (18%; 95% CI, 0.11–0.27). On the other hand, Cao et al. [56] reported that the combination of anti-PD-1 antibody and TKI produced the highest ORR (28%; 95% CI, 0.18–0.40) but was also associated with the highest severe complications (39%; 95% CI, 0.30–0.49). Another approach to enhancing tumor immunogenicity is combining RT with ICIs, although no results from published meta-analyses are currently available. Accordingly, we conducted a narrative review and identified 12 case reports and three clinical studies investigating the combined use of RT and ICI in STS. However, caution is warranted when interpreting these findings, as most cases were unplanned combinations of RT and ICI administered incidentally, often alongside additional modalities such as chemotherapy or surgery. Various RT fractionation schemes, including SBRT, were employed in the case reports, whereas conventional fractionation schemes were used in the clinical studies. Eleven case reports showed promising outcomes for the combination of RT and ICI in STS, although one case report involving LPS suggested the possibility of hyperprogression. Hyperprogression refers to an unexpectedly rapid and aggressive progression of the tumor, with a dramatic acceleration of the disease in immunotherapy, and the incidence ranged from 4% to 30% [57]. The definitions of hyperprogression vary slightly across studies: (1) the first evaluation with at least a twofold increase in tumor growth rate before and after immunotherapy; (2) a >50% increase in tumor burden within less than two month; or (3) a >50% disease progression at the time of the first evaluation compared with baseline prior to treatment [58,59,60]. Although the reported case did not fully meet the established definition of hyperpregression, the authors emphasized that frequent serial imaging is essential for the early detection and monitoring of this rare but clinically significant phenomenon in patients with STS [40].

Ng et al. [46] conducted a phase 1/2 study investigating neoadjuvant tremelimumab and durvalumab combined with RT (50 Gy in 25–28 fractions) followed by surgery and adjuvant durvalumab, in 18 patients with high-risk STS of the trunk and extremities. Histological, radiologic, and clinical outcomes were comparable to those reported in historical cohorts treated without immunotherapy, although the new regimen was well tolerated. In contrast, a randomized, non-comparative phase 2 trial evaluated neoadjuvant nivolumab versus nivolumab combined with ipilimumab in patients with dedifferentiated liposarcoma (DDLPS) and UPS [47]. Among these, neoadjuvant RT (50 Gy in 25 fractions) was administered to UPS patients. The primary endpoint, pathologic response (measured as percentage hyalinization), was 9% in DDPS and 89% in UPS. Two-year DFS and OS rates for UPS were 78% and 90%, respectively. The authors concluded that neoadjuvant ICI therapy combined with concurrent RT demonstrated significant efficacy in UPS. The SU2C-SARC032 phase 2 randomized trial compared neoadjuvant RT followed by surgery (control arm) with neoadjuvant pembrolizumab plus RT (50 Gy in 25 fractions), followed by surgery and adjuvant pembrolizumab (experimental arm), in 143 patients with grade 2 or 3 UPS or DDLPS or pleomorphic LPS [45]. At a median follow-up of 43 months, a modified intention-to-treat analysis of 127 patients revealed that the addition of pembrolizumab significantly improved DFS (hazard ratio, 0.61; 95% CI, 0.39–0.96; *p* = 0.04), although there was no difference in LRFS or OS. Severe complications ≥ grade 3 occurred more frequently in the experimental arm (56%) than in the control arm (31%); however, the toxicity profile of anti-PD-1 therapy was more favorable than that of standard doxorubicin-based combination chemotherapy. A pooled analysis using individual patient data from all prospective trials of ICIs submitted to the US FDA up to 2019 demonstrated that the administration of ICIs within 90 days following RT did not appear to increase the risk of severe complications [61]. These findings suggest that combining RT with ICI therapy may enhance treatment efficacy while maintaining an acceptable safety profile; however, optimal treatment strategies should be validated in future prospective clinical trials.

## 5. Conclusions

The current study demonstrated that combined treatment with anti-PD-1 antibody and RT produced the greatest inhibition of tumor growth and exhibited a synergistic effect in FSaII fibrosarcoma. The accompanying narrative review suggests that combining RT with ICIs in patients with STS may enhance the efficacy of ICIs through synergistic mechanisms while maintaining acceptable toxicity levels. However, given the possibility of hyperprogression and the absence of addictive effects observed with certain ICI regimens, further clinical investigations are warranted to define optimal indications and establish the most effective treatment protocols for STS. The SU2C-SARC032 trial provides us with some answers [45]. The design of this study was evaluated and validated in a preclinical model [49]. Translation research bridging preclinical and clinical findings will be helpful to facilitate the successful implementation of these combined therapeutic strategies in the future.

## Figures and Tables

**Table 1:** Summary of case reports.

Author	Age/Sex	Disease	Treatment	Severe Cx ≥ Gr3
Kalofonou, 2024 [33]	46/M	Metastatic myxofibrosarcoma of extremity	Palliative RT to spine 8 Gy/1 fx followed by ipilimumab/nivolumab for 3 months → PR: nivolumab monotherapy for 11 months → SD and surveillance without treatment	No
Reuben, 2024 [34]	57/M	Metastatic undifferentiated pleomorphic sarcoma of extremity	Pembrolizumab → PR after 3 months → mixed response: local RT to recurrent lesion in thigh 39 Gy and SBRT to oligoprogressive lung mets 50 Gy/5 fx’s → continuing pembrolizumab for 6.5 years: SD	No
Shah, 2023 [35]	53/F	Recurrent undifferentiated pleomorphic sarcoma of heart	Salvage SBRT (re_RT) to recurrent cardiac lesion 25 Gy/1 fx + PD1 inhibitor → PD and death after 16 months without recurrence of primary cardiac lesion	No
Jeon, 2023 [36]	69/F	Primary undifferentiated pleomorphic sarcoma of extremity	Misdiagnosed with melanoma at axilla: ipilimumab/nivolumab 1 cycle → PD with 17 cm-sized mass: re-biopsy which was diagnosed with undifferentiated pleomorphic sarcoma → palliative SBRT 24 Gy/3 fx’s → PR: doxorubicin 6 cycles with neoadjuvant intent → amputation (pathologic CR) → NED at 32 months after initial diagnosis	No
Huang, 2022 [37]	31/M	Primary undifferentiated small round cell sarcoma of lung	VAC + pembrolizumab 6 cycles for 11 cm sized lung mass and mediastinal LNs → PR: radical RT to lung 66 Gy/33 fx’s → VAC + pembrolizumab 2 cycles → continuing pembrolizumab: SD at 14 months after initial diagnosis	No
Damante, 2021 [38]	29/M	Metastatic alveolar soft part sarcoma of extremity	Neoadjuvant SBRT to 3 brain mets 24 Gy/3 fx’s followed by surgery of dominant brain mets → atezolizumab 7 cycles → PR: resection of primary thigh lesion and femur mets → continuing atezolizumab: NED at 15 months after initial diagnosis	NR
Xu, 2021 [39]	32/M	Metastatic alveolar soft part sarcoma of extremity	Local RT to single brain mets 60 Gy/30 fx’s + apatinib/camrelizumab → continuing apatinib/camrelizumab: PR at 6 months after RT	No
Chan, 2020 [40]	63/M	Primary myxoid/round cell liposarcoma of extremity	Neoadjuvant durvalumab + tremelimumab + RT to 13 cm-sized primary mass 15 Gy/1 fx ^a^ followed by 50.4 Gy → surgery (60% necrosis) → PD: newly developed 2 liver mets after 1 month → hyperprogression of liver mets during 3 months → referred to hospice care	NR
Klein, 2019 [41]	88/M	Recurrent pleomorphic dermal sarcoma	Pembrolizumab + salvage RT to recurrent skin lesion 70 Gy/35 fx’s → CR at 4 months	NR
Cramer, 2019 [42]	25/F	Metastatic alveolar soft part sarcoma	SBRT (re_RT) to spine 30 Gy/5 fx’s followed by pembrolizumab → CR at 18 months	NR
Guram, 2018 [43]	51/M	Recurrent undifferentiated pleomorphic sarcoma of maxillary sinus	Nivolumabd + palliative RT to LN mets at neck 30 Gy/10 fx’s → CR at 6 months → marginal recurrence at 13 months → ipilimumab + nivolumab + SBRT to mediastinal LN 24 Gy/3 fx’s → CR at 5 months: NED at 2 years after initial treatment	No
Marcrom, 2017 [44]	26/F	Recurrent clear cell sarcoma of mediastinum	Pembrolizumab + salvage re_RT to chest wall 66 Gy/33 fx’s → CR at 1 month → continuing pembrolizumab	No

Cx, complication; M, male; F, female; RT, radiotherapy; fx, fraction; PR, partial response; SD, stable disease; SBRT, stereotactic body radiotherapy; re_RT, re-irradiation; PD, progressive disease; CR, complete response; NED, no evidence of disease; VAC, vincristine sulfate, dactinomycin, and cyclophosphamide chemotherapy; LN, lymph node; mets, metastasis; NR, not reported. ^a^ A single 15 Gy fraction of spatially fractionated GRID radiation therapy was delivered to the gross tumor volume.

**Table 2:** Summary of clinical trials.

Author	Study Design	No. of Pts	Histology (%)	Primary Tumor Location (%)	Disease Status (%)	Treatment	LR	DFS	OS	Severe Cx
Mowery, 2024 [45]	Phase 2	64	DDPS (6), myxofibrosarcoma (11), UPS (83)	Extremity (100)	Primary (100)	Neoadjuvant pembrolizumab + RT (50 Gy/25 fx’s) → surgery → pembrolizumab 14 cycles	3%	67% at 2 years	88% at 2 years	56%
		63	DDPS (6), Pleomorphic LPS (8), myxofibrosarcoma (10), UPS (76)	Extremity (100)	Primary (100)	Neoadjuvant RT (50 Gy/25 fx’s) → surgery	0%	52% at 2 years	85% at 2 years	31%
Ng, 2024 [46]	Phase 1/2	18	Myxofibrosarcoma (11), LPS (28), synovial sarcoma (6), UPS (50), unclassified (5)	Extremity (100)	Primary (89) Recurrent (11)	Neoadjuvant tremelimumab/durvalumab 3 cycles + RT (50 Gy/25-28 fx’s ± SFGRT ^a^) → surgery → durvalumab 4 cycles	11%			17%
Roland, 2024 [47]	Phase 2	10	UPS (100)	Extremity/trunk (100)	Primary (90) Recurrent (10)	Neoadjuvant nivolumab + RT (50 Gy/25 fx’s) → surgery (60)	0%	60% at 2 years	83% at 2 years	26% ^b^
						Neoadjuvant nivolumab/ipilimumab + RT (50 Gy/25 fx’s) → surgery (40)	0%	100% at 2 years	100% at 2 years	

DDPS, dedifferentiated liposarcoma; UPS, undifferentiated pleomorphic sarcoma; LPS, liposarcoma; RT, radiotherapy; fx, fraction; LR, local recurrence; DFS, disease-free survival; OS, overall survival; Cx, complication. ^a^ A single 15 Gy fraction of spatially fractionated GRID radiation therapy was delivered to the gross tumor volume within one to three days prior to the start of 50 Gy/25-28 fractions for bulky sarcoma, defined >10 cm in greatest dimension. ^b^ Severe complications occurred in 7 out of 27 patients with retroperitoneal DDPS (n = 17) treated with immunotherapy + surgery and extremity/trunk UPS (n = 10) treated with immunotherapy + RT + surgery.

## Data Availability

The data presented in this study are available on request from the corresponding author.
